# A Rare Presentation of Concomitant Lung Disease and Hepatitis After Rituximab Treatment: A Case Report

**DOI:** 10.7759/cureus.38910

**Published:** 2023-05-11

**Authors:** Linda Albusoul, Hussna Abunafeesa, Vrushali Dabak

**Affiliations:** 1 Internal Medicine, Henry Ford Health System, Detroit, USA; 2 Hematology and Oncology, Henry Ford Health System, Detroit, USA

**Keywords:** interstitial lung disease, rituximab induced hepatitis, follicular lymphoma, rituximab induced lung disease, rituximab therapy

## Abstract

Rituximab (RTX) is a chimeric monoclonal antibody that is a standard component of treatment for all B-cell malignancies. The most common adverse events related to RTX are infusion-related reactions, such as fever, chills, urticaria, flushing, and headaches. However, RTX-induced lung disease (RTX-ILD) is a rare but potentially fatal adverse reaction, and diagnosing RTX-ILD is challenging, especially when accompanied by other rare adverse reactions, such as hepatitis. Here, we report a case of RTX-ILD with concomitant RTX-induced hepatitis in a 55-year-old man with follicular B-cell non-Hodgkin lymphoma who was on maintenance RTX therapy. The patient presented with a subacute, persistent dry cough, shortness of breath, fevers, and chills shortly after having traveled. Outpatient antibiotic therapy did not relieve symptoms, and laboratory studies revealed evidence of liver injury. A computed tomography (CT) of the chest showed predominately basilar airspace disease and ground glass opacities suggestive of multifocal pneumonia. Extensive infectious and autoimmune workups were negative. RTX-ILD with concomitant RTX-induced hepatitis was considered because antibiotic therapy did not resolve symptoms or improve signs of liver damage. Prednisone (1 mg/kg) led to symptom resolution and liver enzyme improvement. The patient underwent a 30-day steroid taper and the withholding of RTX infusions. A CT of the chest three months after discharge showed nearly resolved multifocal ground glass opacities. RTX-ILD should be considered after infectious and autoimmune etiologies have been ruled out for all patients on RTX therapy who experience symptoms of lung pathology or infection.

## Introduction

Rituximab (RTX) is a chimeric human-murine immunoglobin G1-kappa monoclonal antibody therapy with a specific affinity for the CD20 transmembrane protein on B lymphocytes. CD20 is expressed on normal B cells, excluding stem and plasma cells, and it is also expressed on most malignant B-cells [1,2]. There are at least four described mechanisms by which RTX eliminates cells that express CD20, including antibody-mediated cellular cytotoxicity, antibody-mediated cellular phagocytosis, complement-dependent cytotoxicity, and direct antitumor effects via apoptosis [2]. 

Intravenous RTX was approved by the US Food and Drug Administration in 1997 and by the European Agency for the Evaluation of Medicinal Products in 1998 for low-grade non-Hodgkin lymphoma. Approvals for using RTX to treat chronic lymphocytic leukemia and diffuse large B-cell non-Hodgkin lymphoma followed [1,2]. RTX was the first therapeutic monoclonal antibody used in oncology, and it was a major forward step in the treatment of B-cell malignancies, becoming a standard component of therapy for follicular lymphoma, chronic lymphocytic leukemia, and diffuse large B-cell non-Hodgkin lymphoma [1,2]. Since then, indications for RTX have been extended to other hematological malignancies as well as non-hematological conditions, such as rheumatoid arthritis and granulomatosis with polyangiitis [2]. 

RTX has a well-established safety profile, both as monotherapy and in combination with other chemotherapeutic drugs. However, RTX-induced lung disease (RTX-ILD) is a rare adverse reaction [1,3]. Additionally, RTX-induced hepatitis is a rare adverse reaction. The presentation of two concomitant adverse drug reactions is particularly unusual and can be challenging to diagnose. 

The purpose of this case report is to outline the process of diagnosis and successful treatment of a man who was being treated with RTX for follicular B-cell non-Hodgkin lymphoma and who developed concomitant RTX-ILD and RTX-induced hepatitis. The rarity of this combination of adverse events from a well-established and safe cancer therapy highlights the importance of considering RTX-induced pathology when infectious and autoimmune disorders have been excluded in patients who present with symptoms of lung pathology and liver damage. 

## Case presentation

A 55-year-old man presented to the Emergency Department with a persistent dry cough, shortness of breath, fevers, and chills of three weeks duration. He had a medical history of smoldering multiple myeloma and follicular B-cell non-Hodgkin lymphoma; his status was post-six cycles of combination bendamustine-RTX and five cycles of maintenance RTX. 

One month before his presentation, the patient had traveled to Colorado for a hiking trip and subsequently developed a cough and myalgias. He tested positive for COVID-19, but he was not initiated on treatment because he was outside the time window. The patient’s COVID-19 symptoms resolved, and he was able to receive his scheduled RTX infusion 27 days after his positive COVID-19 test. He then began to experience a dry cough, shortness of breath, fevers, and chills one week after his RTX infusion (the 11th cycle) and was evaluated by his primary care physician. Radiography of the chest showed multifocal pneumonia. He was subsequently treated with two courses of outpatient antibiotics, but his symptoms persisted, prompting him to present to the Emergency Department. 

**Figure 1:**
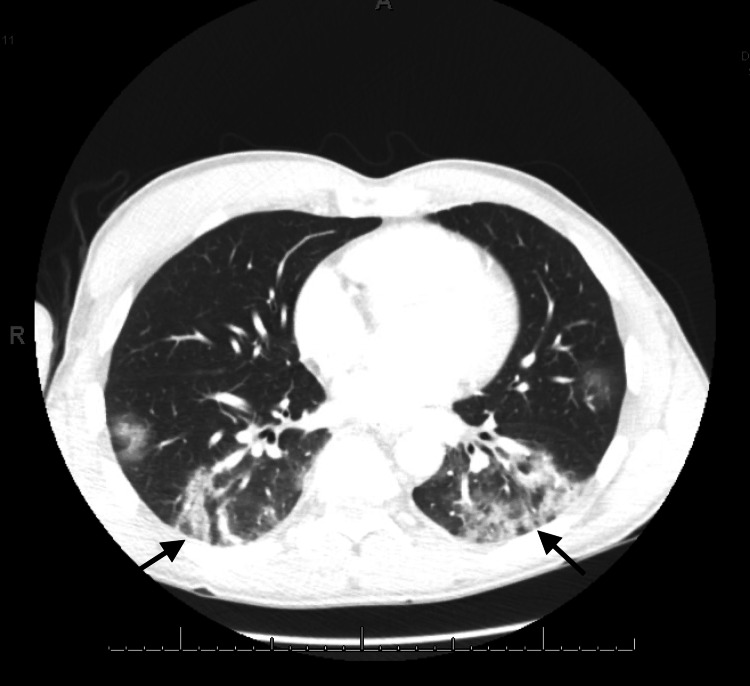
Basilar’s predominating patchy airspace and ground-glass opacities

The patient’s vital signs on presentation were as follows: temperature: 39.4°C, blood pressure 129/66, heart rate 83 beats/minute, respiratory rate 20 breaths/minute, and oxygen saturation 98% on room air. Physical examination revealed bilateral coarse lung sounds. Laboratory test results showed elevated liver enzymes, including alanine transaminase (ALT) at 243 IU/L (reference range [RR] <52 IU/L), aspartate transaminase (AST) at 146 IU/L (RR <35 IU/L), and alkaline phosphatase (ALP) at 671 IU/L (RR 40-140 IU/L), and normal total bilirubin of 1 mg/dL (RR < 1.2 mg/dL). A computed tomography (CT) of the chest showed predominately basilar patchy airspace disease and ground-glass opacities suggestive of multifocal pneumonia or drug-induced pneumonitis, as demonstrated in Figure 1. Ultrasonographic imaging of the abdomen showed mildly coarse hepatic echotexture suggestive of hepatitis. 

The considered differential diagnoses included infectious, inflammatory, autoimmune, or drug-induced processes. The infectious disease service was consulted, given the patient’s immunosuppressed status and significant travel history. An extensive work-up for typical and atypical infectious organisms revealed no positive results. A repeat COVID-19 test was also negative. Results of an autoimmune work-up that included antinuclear antibody and neutrophilic cytoplasmic antibody titers were negative. Regarding elevated liver enzymes, a hepatitis screen and an autoimmune workup were both negative. The patient then underwent bronchoscopy with bronchoalveolar lavage (BAL). Respiratory cultures from the BAL sample grew alpha-hemolytic Streptococcus species. BAL pathology showed lymphoid cells; however, an insufficient quantity precluded further assessment. Notably, BAL analysis was also negative for malignant cells. 

**Figure 2:**
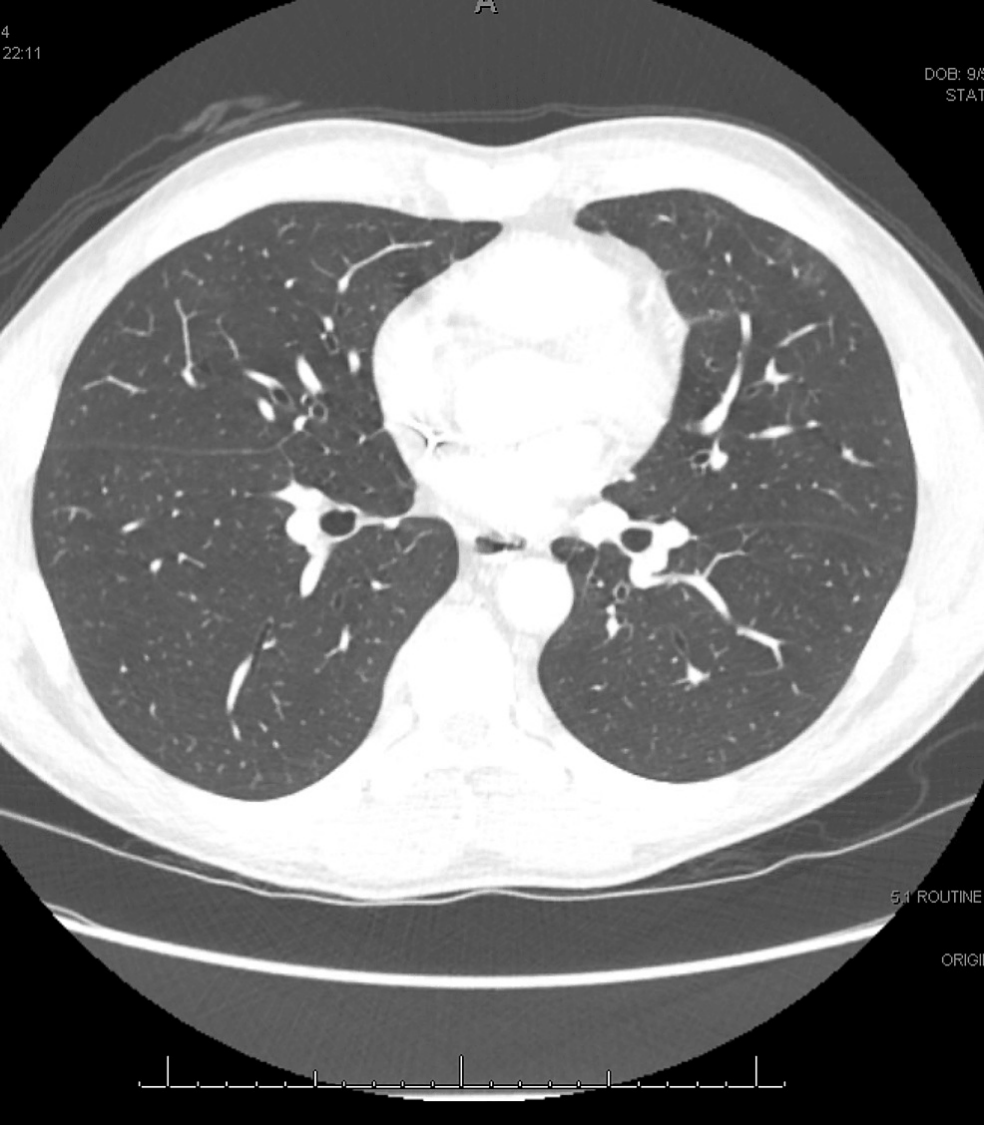
Nearly resolved multifocal ground-glass airspace disease

Our patient received a 10-day course of ceftriaxone and doxycycline to empirically cover for possible Rickettsial disease until finalization of cultures. Despite treatment, he continued to have persistent fever and chills, and his liver enzymes continued to increase daily. He was subsequently started on prednisone 1 mg/kg for suspected RTX-induced lung disease and hepatitis, following the exclusion of other etiologies. His fever then resolved, and he had dramatic symptomatic improvement. Additionally, his liver enzymes began to improve. His most recent laboratory tests showed a return to normal AST and ALT levels and a significant decrease in ALP. Throughout his hospital stay, he became hypoxic to 91% on room air but did not require supplemental oxygen. He was continued on high-dose prednisone with a plan for a 30-day taper. He was also started on trimethoprim-sulfamethoxazole while on steroid therapy for Pneumocystis jirovecii prophylaxis. Repeat radiography of the chest showed minimal residual basilar opacity, and CT of the chest before discharge showed diffuse ground-glass opacities greatest in the bases. The interpretation of the findings was consistent with a drug-induced pneumonitis with ongoing consideration for other possibilities, such as an atypical infection. He was followed closely with weekly outpatient visits, where he reported sustained resolution of cough and fever while steroids were tapered. His RTX treatment was put on hold. A repeat CT of the chest three months after discharge showed nearly resolved multifocal ground glass opacities, as demonstrated in Figure 2. The current plan is to repeat the CT of the chest before further treatment decisions. 

## Discussion

The RTX safety profile, both as a monotherapy and in combination with other chemotherapeutics, has been well established with over 20 years of post-marketing surveillance and more than four million patient exposures [2]. The most common adverse drug reactions related to RTX are infusion-related reactions, including but not limited to fever, chills, urticaria, flushing, and headaches. Infusion-related reactions of any grade have been reported in 77% of patients treated with RTX; however, more severe reactions causing bronchospasm or hypotension have been reported in 12% of patients [2]. Infusion-related reactions are more common following the first infusion, and the frequency decreases with subsequent infusions [2]. 

Adverse respiratory systems manifestations such as cough, dyspnea, rhinitis, and sinusitis have been reported in up to 38% of patients on RTX [3], and the incidence of RTX-related delayed lung injury is in the range of 0.01% to 0.03% [1,3]. Different patterns of RTX-related lung injury have been described. A systematic review that assessed 52 cases of RTX-ILD found that the most common presentation was acute or subacute organizing pneumonia [1]. More rarely, acute respiratory distress syndrome and diffuse alveolar hemorrhage have been noted [1,3,4]. The review by Liote et al. additionally noted that the mean time of onset of respiratory symptoms was 12 weeks following initiation of RTX therapy, with a peak after the fourth cycle or at a cumulative dose of 1,600 mg/m2 [1]. Another systematic review that considered 121 cases of RTX-ILD reported cases developing as early as after the first cycle and as late as after the 12^th^ cycle of RTX, with a median onset of 15 days after the last infusion [3]. In the case of our patient, he developed respiratory symptoms one week following the 11^th^ cycle of RTX infusion, or at a cumulative dose of 4125 mg/m2, which puts him at the late end of the spectrum. 

The median age of patients who are diagnosed with RTX-ILD is approximately 65 years, and this adverse event is more common in men than in women [1]. The most common presenting symptoms of RTX-ILD are fever, dyspnea, and dry cough [3]. Radiological findings in cases of RTX-ILD are bilateral and diffuse in most patients, similar to our patient’s presentation [1,3]. Interestingly, some patients on RTX have had radiological abnormalities in the lungs without having any overt symptoms [3]. Fluorodeoxyglucose positron emission tomography-CT is a very sensitive modality for identifying RTX-ILD, and this approach can detect early disease [3-5]. 

The mechanism underlying RTX-ILD is still not well understood; however, multiple mechanisms that could contribute to lung injury have been proposed, including cytotoxic T lymphocyte dysregulation due to prolonged B-cell depletion, leuco-stasis in the pulmonary circulation, the release of inflammatory cytokines, and tumor lysis syndrome [3,5,6]. 

RTX-ILD is a diagnosis of exclusion, and other differential diagnoses need to be ruled out, including infection and autoimmune processes such as vasculitis and the progression of the underlying disease. In our patient, an extensive infectious work-up was done, which included blood cultures, BAL fluid cultures (bacterial, viral, and fungal), a respiratory pathogen panel test, rickettsia antibody testing, and testing for atypical organisms, which was unremarkable. The patient’s recent history of COVID-19 infection before his presentation further complicated the diagnosis; nevertheless, his symptoms completely resolved before he received his 11^th^ RTX infusion, and his repeat COVID-19 testing was negative. Our patient’s BAL fluid bacterial cultures were positive for alpha-hemolytic Streptococcus species; however, our patient was treated with multiple courses of antibiotics, both outpatient and inpatient and yet his symptoms persisted, which raised concern for the presence of a commensal bacterium. One retrospective study of infectious agents in BAL fluid from patients with hematological malignancies and pulmonary infiltrates reported that alpha-hemolytic Streptococcus was one of the most common bacteria found in BAL samples; however, the significance of this organism remains unclear since it is a common commensal organism that does not frequently cause infection [7]. Furthermore, our patient’s autoimmune work-up was negative, and the BAL fluid cytology did not show any malignant cells. After the common causes of pulmonary infiltrates were excluded, RTX-ILD was considered, and the patient was initiated on appropriate steroid therapy and had a favorable response. 

An added layer of complexity to this case was the concomitant liver injury that our patient experienced. RTX has been noted to reactivate the hepatitis B virus [2,8,9], and two cases of RTX-induced hepatitis have been reported [8,9]. In our patient, both infectious and autoimmune workups were unremarkable. And while ultrasonography of the abdomen was concerning for hepatitis, it did not reveal any other intra-abdominal pathology that could explain the rise in liver enzymes. Thus, although our patient’s liver enzymes continued to increase, they slowly and steadily started to decrease shortly after the initiation of steroid therapy. 

## Conclusions

In conclusion, for any patient receiving RTX therapy who presents with respiratory symptoms, regardless of the cycle or timing following the last infusion, a diagnosis of RTX-ILD should be considered following the exclusion of more common infectious and autoimmune etiologies. Although RTX-ILD is a rare adverse event, it can potentially be fatal. The presentation of two concomitant adverse drug reactions is particularly rare and can pose a diagnostic challenge; however, clinicians should keep RTX-ILD high on the differential diagnoses for patients on RTX so that treatment can be initiated promptly before further lung or liver damage occurs. 
